# Cardiac magnetic resonance imaging in healthy volunteers in normoxic and hypoxic exercise

**DOI:** 10.1186/1532-429X-18-S1-Q22

**Published:** 2016-01-27

**Authors:** Shareen Jaijee, Marina Quinlan, Pawel F Tokarczuk, Benjamin Statton, Tamara Diamond, Luke Howard, John Gibbs, Declan P O'Regan

**Affiliations:** 1 Medical Research Council, London, United Kingdom; 2 Imperial College London, London, United Kingdom; 3 National Institute Heart and Lung Research, London, United Kingdom

## Background

Hypoxia induces pulmonary vasoconstriction leading to an increase in pulmonary vascular resistance and acute pulmonary hypertension (PH). It is known that with acute hypoxia, cardiac output (CO) is maintained with an increase in heart rate (HR) but a blunted stroke volume (SV) response. To our knowledge, the response of the healthy heart to acute pulmonary hypertension has not been studied using exercise cardiac magnetic resonance imaging (CMR). CMR is a highly reproducible and accurate method of evaluating biventricular function during exercise and can be used to measure real time physiological response to an increased cardiovascular workload. We aimed to study the ability of exercise magnetic resonance imaging to detect changes in right ventricular (RV) and left ventricular (LV) function during normoxic and hypoxic exercise.

## Methods

Resting and exercise CMR breathing room air and 12% oxygen was carried out on 6 healthy volunteers (males = 3 ,average age = 39 years), using a Philips 1.5T Achieva (Best, Netherlands) with a 32-channel cardiac coil. Supine exercise was performed using an MR compatible ergometer (Lode, Netherlands) at 40% of the watts achieved on cardiopulmonary exercise testing. High temporal resolution free breathing real-time phase contrast imaging of the aorta were acquired. Automated correction of voxel aliasing was performed in Matlab if required. Average stroke volume (SV) and cardiac output (CO) were calculated over a 5 second period using Art Fun (Inserm, Paris) with automated in-plane vessel tracking. CO was calculated as SV × heart rate (HR). The Wilcoxon signed rank test was used to compare resting and exercise values, during normoxic and hypoxic exercise.

## Results

All subjects tolerated both the normoxic and hypoxic rest and exercise CMR and were able to sustain their target power output. Median peripheral oxygen saturation decreased from 98% at to 87.5% at rest and 79.5% on exercise At rest, there was no significant difference between SV during normoxia and hypoxia (97+/-32 mL vs 105+/-41 mL), however, there was a significant increase in resting HR (57+/-9 vs 69+/-10 bpm) (p = 0.03) and CO (5.2+/-1.4 L/min vs 6.5+/-1.9 L/min) (p = 0.03). During normoxic exercise, there was a significant increase in HR to 116+/-14 bpm, SV to 126+/-39 mL and CO to 15+/- 6 L/min (p = 0.03). During hypoxic exercise, there was a significant increase in HR to 135+/-13 bpm and CO to 16+/-5.5 L/min (p = 0.03), with a higher exercise HR than normoxic exercise (p = 0.03). However, there was no increase in SV (105+/-41 mL to 118+/-36 mL).

## Conclusions

During normobaric hypoxia in healthy volunteers, SV changes become apparent on exercise which are otherwise not detectable at rest. This could potentially be valuable clinically in the assessment and prognostication in heart failure and pulmonary hypertension where reduced exercise capacity limits quality of life.

